# Author Correction: Chromosomal scale assembly of parasitic wasp genome reveals symbiotic virus colonization

**DOI:** 10.1038/s42003-021-02480-9

**Published:** 2021-07-30

**Authors:** Jérémy Gauthier, Hélène Boulain, Joke J. F. A. van Vugt, Lyam Baudry, Emma Persyn, Jean-Marc Aury, Benjamin Noel, Anthony Bretaudeau, Fabrice Legeai, Sven Warris, Mohamed A. Chebbi, Géraldine Dubreuil, Bernard Duvic, Natacha Kremer, Philippe Gayral, Karine Musset, Thibaut Josse, Diane Bigot, Christophe Bressac, Sébastien Moreau, Georges Periquet, Myriam Harry, Nicolas Montagné, Isabelle Boulogne, Mahnaz Sabeti-Azad, Martine Maïbèche, Thomas Chertemps, Frédérique Hilliou, David Siaussat, Joëlle Amselem, Isabelle Luyten, Claire Capdevielle-Dulac, Karine Labadie, Bruna Laís Merlin, Valérie Barbe, Jetske G. de Boer, Martial Marbouty, Fernando Luis Cônsoli, Stéphane Dupas, Aurélie Hua-Van, Gaelle Le Goff, Annie Bézier, Emmanuelle Jacquin-Joly, James B. Whitfield, Louise E. M. Vet, Hans M. Smid, Laure Kaiser, Romain Koszul, Elisabeth Huguet, Elisabeth A. Herniou, Jean-Michel Drezen

**Affiliations:** 1 Institut de Recherche sur la Biologie de l’Insecte, UMR 7261 CNRS-Université de Tours, Faculté des Sciences et Techniques, Parc de Grandmont, 37200 Tours, France; 2 Geneva Natural History Museum, 1208 Geneva, Switzerland; 3 EAWAG, Swiss Federal Institute of Aquatic Science and Technology, Dübendorf, Switzerland; 4 Department of Terrestrial Ecology, Netherlands Institute of Ecology (NIOO-KNAW), Droevendaalsesteeg 10, 6708 PB Wageningen, The Netherlands; 5 Institut Pasteur, Unité Régulation Spatiale des Génomes, UMR 3525, CNRS, Paris, 75015 France; 6 Sorbonne Université, Collège Doctoral, 75005 Paris, France; 7 Sorbonne Université, INRAE, CNRS, IRD, UPEC, Univ. de Paris, Institute of Ecology and Environmental Science of Paris (iEES-Paris), 75005 Paris, France; 8 Génomique Métabolique, Genoscope, Institut François Jacob, CEA, CNRS, Univ Evry, Université Paris-Saclay, 91057 Evry, France; 9 IGEPP, INRAE, Institut Agro, Univ Rennes, 35000 Rennes, France; 10 Univ Rennes, Inria, CNRS, IRISA, 35000 Rennes, France; 11 Applied Bioinformatics, Wageningen University & Research, Wageningen, The Netherlands; 12 Université Montpellier, INRAE, DGIMI, 34095 Montpellier, France; 13 Laboratoire de Biométrie et Biologie Evolutive Université de Lyon, Université Claude Bernard Lyon 1, CNRS, UMR 5558, 43 bd du 11 novembre 1918, bat. G. Mendel, 69622 Villeurbanne Cedex, France; 14 Université Paris-Saclay, CNRS, IRD, UMR Évolution, Génomes, Comportement et Écologie, 91198 Gif-sur-Yvette, France; 15 Université Côte d’Azur, INRAE, CNRS, ISA, 06903 Sophia-Antipolis, France; 16 Université Paris-Saclay, INRAE, URGI, 78026 Versailles, France; 17 Insect Interactions Laboratory, Department of Entomology and Acarology, Luiz de Queiroz College of Agriculture (ESALQ), University of São Paulo, Piracicaba, São Paulo 13418-900 Brazil; 18 Laboratory of Entomology, Wageningen University, P.O. Box 16, Droevendaalsesteeg 1, 6708 PB Wageningen, The Netherlands; 19 Evolutionary Genetics, University of Groningen, Nijenborgh 4, 9747 AG Groningen, The Netherlands; 20 Department of Entomology, 320 Morrill Hall, 505 South Goodwin Avenue, University of Illinois, Urbana, IL 61801 USA

**Keywords:** Genome evolution, Evolutionary genetics, Entomology

Correction to: *Communications Biology* 10.1038/s42003-020-01623-8, published online 22 January 2021.

In the original version of the Article, the project accession codes for the genome assembly and for the umbrella project were omitted. The Data availability statement should read “The datasets and genomes generated during the current study are available from the European Bioinformatic Institute (EMBL-EBI) and National Center for Biotechnology Information (NCBI) at the following BioProject IDs: PRJEB36310 and PRJEB43234 (umbrella project PRJEB40240). Genome database (genomes and annotated genes) is also available on the web site BIPAA (Bioinformatic Platform for Agrosystem Arthropods) https://bipaa.genouest.org/is/parwaspdb/”.

This has now been corrected in the PDF and HTML versions of the Article.

