# Supplementary material for: Chromosomal scale assembly of parasitic wasp genome reveals symbiotic virus colonization
Source: Commun Biol. 2021 Jan 22;4:104. doi: 10.1038/s42003-020-01623-8 (PMC7822920; doi:10.1038/s42003-020-01623-8)
Supplement: Supplementary file 2 — Supplementary Information [file 42003_2020_1623_MOESM2_ESM.pdf]

Supplementary Materials for

Chromosomal scale genome assembly reveals symbiotic virus colonization of parasitic wasp DNA

J. Gauthier, H. Boulain, J. J.F.A. van Vugt, L. Baudry, E. Persyn, J.-M. Aury, B. Noel, A. Bretaudeau, F. Legeai, S. Warris, M. A. Chebbi, G. Dubreuil, B. Duvic, N. Kremer, P. Gayral, K. Musset, T. Josse, D. Bigot, C. Bressac, S. Moreau, G. Periquet, M. Harry, N. Montagné, I. Boulogne, M. Sabeti-Azad, M. Maïbèche, T. Chertemps, F. Hilliou, D. Siaussat, J. Amselem, I. Luyten, C. Capdevielle-Dulac, K. Labadie, B. Laís Merlin, V. Barbe, J. G. de Boer, M. Marbouty, F. L. Cônsoli, S. Dupas, A. Hua Van, G. Le Goff, A. Bézier, E. Jacquin-Joly, J. B. Whitfield, L.E.M. Vet, H. M. Smid, L. Kaiser-Arnault, R. Koszul, E. Huguet, E. A. Herniou and J.-M.Drezen*.

*corresponding author: drezen@univ-tours.fr

A

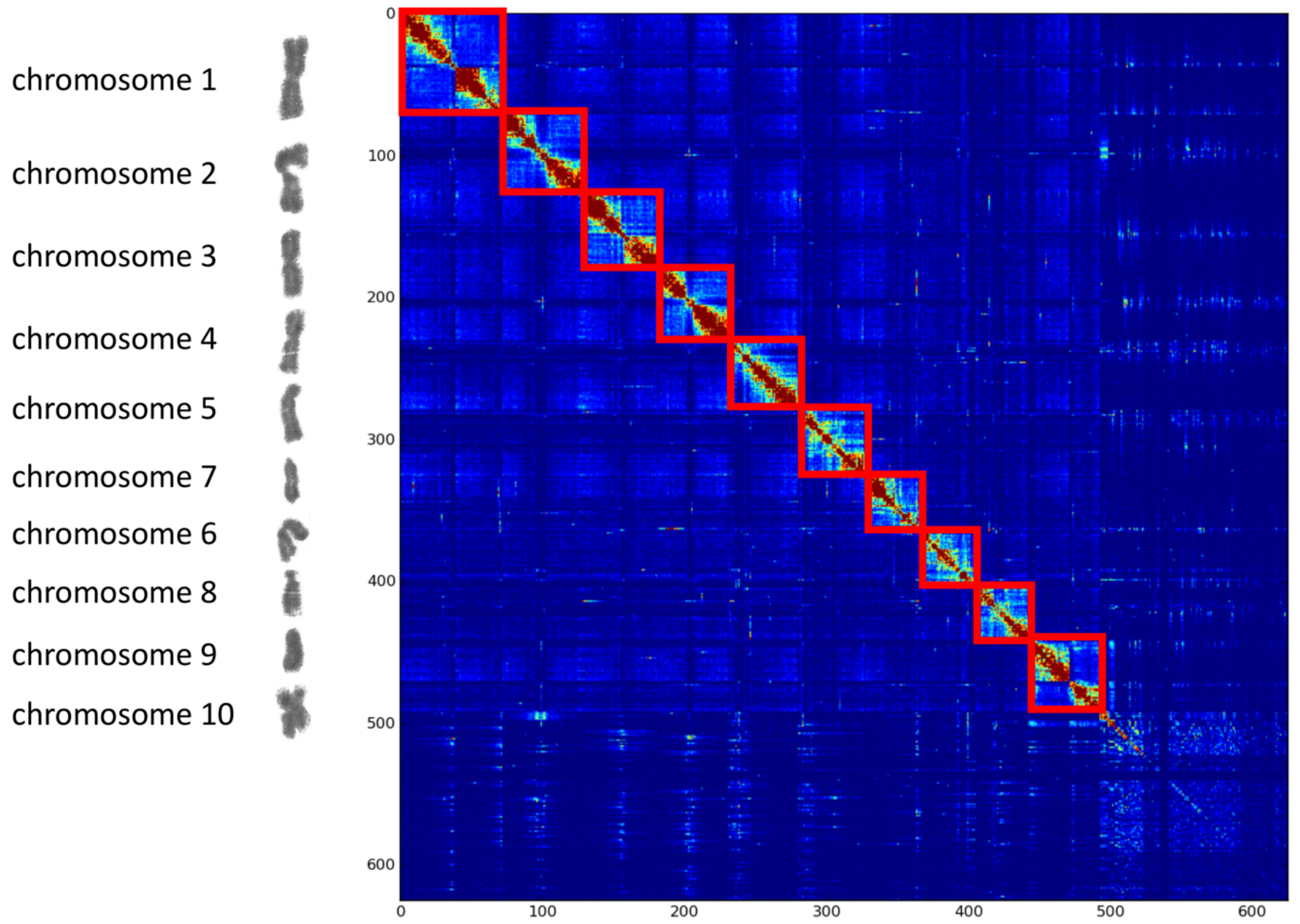
chromosome 1
chromosome 2
chromosome 3
chromosome 4
chromosome 5
chromosome 7
chromosome 6
chromosome 8
chromosome 9
chromosome 10
0
100
200
300
400
500
600

B

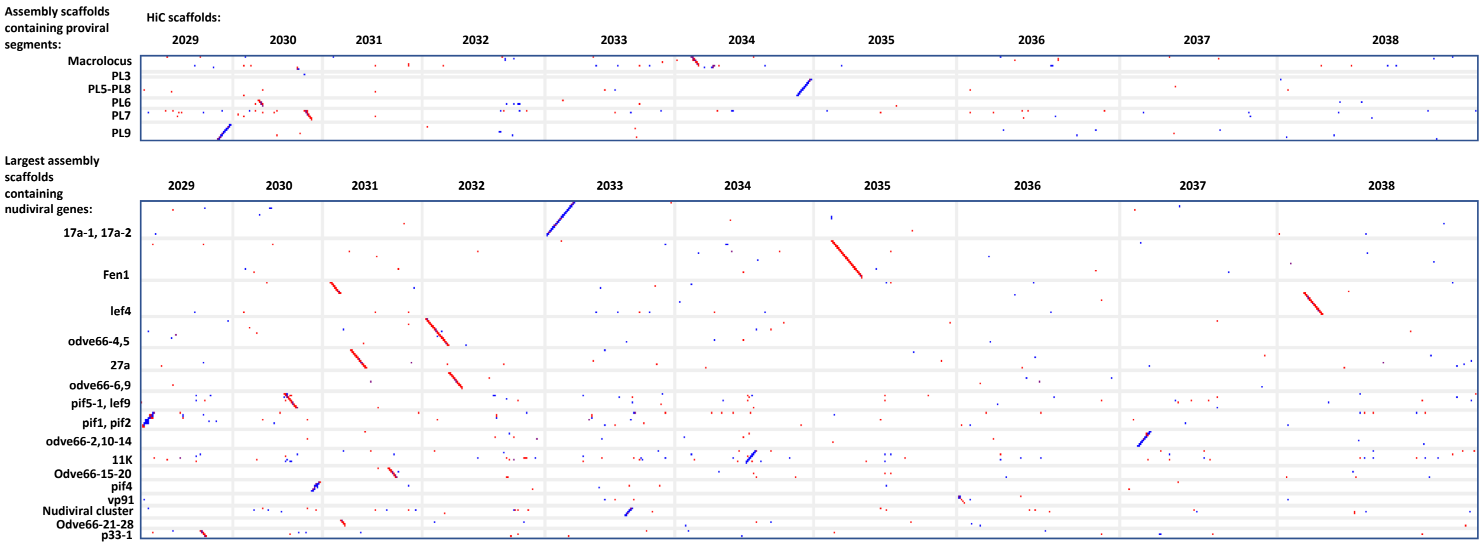
Assembly scaffolds containing proviral segments:
HiC scaffolds:
2029
2030
2031
2032
2033
2034
2035
2036
2037
2038
Macrolocus
PL3
PL5-PL8
PL6
PL7
PL9
Largest assembly scaffolds containing nudiviral genes:
17a-1, 17a-2
Fen1
lef4
odve66-4,5
27a
odve66-6,9
pif5-1, lef9
pif1, pif2
odve66-2,10-14
11K
Odve66-15-20
pif4
vp91
Nudiviral cluster
Odve66-21-28
p33-1

Supplementary Figure 1. A Contact matrix between each fragment used for the GRAAL assembly, from blue the most distant to red the closest fragments. Along the two axis the fragments are organized according to this distance. The red frames have been added to visualize the chromosomes separations. The assignment of each chromosome has been performed using the chromosome length and centromere positions but also including sequences used for *in situ* hybridization performed by Belle and colleagues (doi:10.1128/JVI.76.11.5793-5796.2002) **B** Dot plot: assembly scaffolds containing bracovirus sequences/HiC scaffolds. Assembly scaffolds are confirmed by HiC scaffolds (except for the *lef4*-containing scaffold which is split in two pieces in HiC).

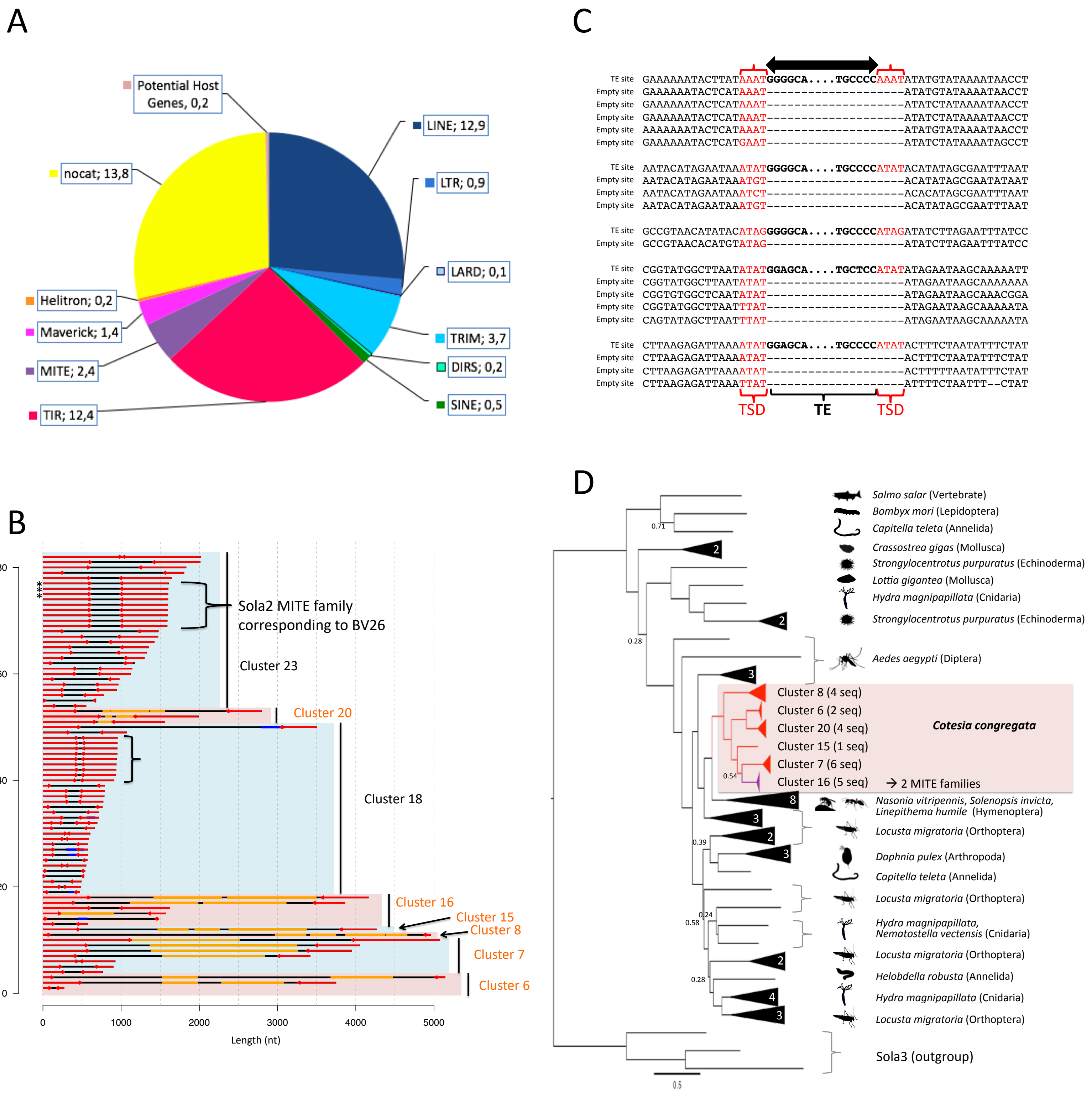
A
Potential Host Genes, 0,2
LINE; 12,9
LTR; 0,9
LARD; 0,1
TRIM; 3,7
DIRS; 0,2
SINE; 0,5
TIR; 12,4
MITE; 2,4
Maverick; 1,4
Helitron; 0,2
nocat; 13,8
C
TE site GAAAAAATACTTATAAATGGGGCA....TGCCCCAAATATATGTATAAAATAACCT
Empty site GAAAAAATACTCATAAAT---------------------ATATGTATAAAATAACCT
Empty site GAAAAAATACTCATAAAT---------------------ATATCTATAAAATAACCT
Empty site GAAAAAATACTCATAAAT---------------------ATATCTATAAAATAACCT
Empty site AAAAAAATACTCATAAAT---------------------ATATGTATAAAATAACCT
Empty site GAAAAAATACTCATGAAT---------------------ATATCTATAAAATAGCCT
TE site AATACATAGAATAAATATGGGGCA....TGCCCCATATACATATAGCGAATTTAAT
Empty site AATACATAGAATAAATGT---------------------ACATATAGCGAATATAAT
Empty site AATACATAGAATAAATCT---------------------ACACATAGCGAATTTAAT
Empty site AATACATAGAATAAATGT---------------------ACATATAGCGAATTTAAT
TE site GCCGTAACATATACATAGGGGGCA....TGCCCCATAGATATCTTAGAATTTATCC
Empty site GCCGTAACACATGTATAG---------------------ATATCTTAGAATTTATCC
TE site CGGTATGGCTTAATATATGGAGCA....TGCTCCATATATAGAATAAGCAAAAATT
Empty site CGGTATGGCTTAATATAT---------------------ATAGAATAAGCAAAAAAA
Empty site CGGTGTGGCTCAATATAT---------------------ATAGAATAAGCAAACGGA
Empty site CGGTATGGCTTAATTTAT---------------------ATAGAATAAGCAAAAATA
Empty site CAGTATAGCTTAATTTAT---------------------ATAGAATAAGCAAAAATA
TE site CTTAAGAGATTAAAATATGGAGCA....TGCCCCATATACTTTCTAATATTTCTAT
Empty site CTTAAGAGATTAAAATAT---------------------ACTTTCTAATATTTCTAT
Empty site CTTAAGAGATTAAAATAT---------------------ACTTTTTAATATTTCTAT
Empty site CTTAAGAGATTAAATTAT---------------------ATTTTCTAATTT--CTAT
TSD
TE
TSD
B
***
Sola2 MITE family corresponding to BV26
Cluster 23
Cluster 20
Cluster 18
Cluster 16
Cluster 15
Cluster 8
Cluster 7
Cluster 6
0
20
40
60
80
0
1000
2000
3000
4000
5000
Length (nt)
D
Salmo salar (Vertebrate)
Bombyx mori (Lepidoptera)
Capitella teleta (Annelida)
Crassostrea gigas (Mollusca)
Strongylocentrotus purpuratus (Echinoderma)
Lottia gigantea (Mollusca)
Hydra magnipapillata (Cnidaria)
Strongylocentrotus purpuratus (Echinoderma)
Aedes aegypti (Diptera)
Cluster 8 (4 seq)
Cluster 6 (2 seq)
Cluster 20 (4 seq)
Cluster 15 (1 seq)
Cluster 7 (6 seq)
Cluster 16 (5 seq)
→ 2 MITE families
Cotesia congregata
Nasonia vitripennis, Solenopsis invicta, Linepithema humile (Hymenoptera)
Locusta migratoria (Orthoptera)
Daphnia pulex (Arthropoda)
Capitella teleta (Annelida)
Locusta migratoria (Orthoptera)
Hydra magnipapillata, Nematostella vectensis (Cnidaria)
Locusta migratoria (Orthoptera)
Helobdella robusta (Annelida)
Hydra magnipapillata (Cnidaria)
Locusta migratoria (Orthoptera)
Sola3 (outgroup)
0.71
0.28
0.54
0.39
0.24
0.58
0.28
0.5
2
2
3
8
3
2
3
2
4
3

Supplementary Figure 2. REPET annotation of transposable elements and characteristics of Sola2 elements at the origin of bracovirus virulence genes BV26. **A** Pie plot of transposable elements organized by families, as detected by REPET. **B** Structure of the Sola2 family. BV26 corresponds to a MITE (Miniature Inverted-repeat Transposable Element), 1606 bp long with 616 nt TIR (Terminal Inverted Repeats) related to the TIR of a Sola2 family (BLASTN). 82 related sequences were grouped into 8 clusters (usearch v5.1 (http://www.drive5.com/usearch) -id=0.8). Six clusters (name in orange) contained sequences with homology to Sola2 transposase (orange part) (blastx against RepBase20.05_REPET edition, -evalue 1e-4). Blue parts indicate homology with other TEs from RepBase. Red arrows represent the TIRs of the elements. The two most abundant correspond to 2 MITE clusters and copies resulting from amplification (same size and sequences) are highlighted by brackets. The asterisks indicates the 3 copies previously annotated as bracovirus genes BV26. **C** Graph showing the target site duplication (4-nt, TA rich) produced during element insertion deduced from the comparison of some Sola2 insertions sites with paralogous empty sites. As for typical Sola2 element, TIRs start by G-rich sequences. **D** Phylogenetic tree showing the relationships between Sola2 translated sequences from *C. congregata* (framed branches) and other Sola2 transposases from RepBase. The alignment (MAFFT v7 (https://mafft.cbrc.jp/alignment/software/)) was truncated to keep only the 250 AA-long best conserved part (AA 240 to 520 of Sola2-1_Nvi) for a phylogenetic analysis using FastTree (http://www.microbesonline.org/fasttree/). Black triangles group TE consensus sequences from the same species (number of sequences is indicated). Open triangles represent consensus sequences of Sola3 elements (RepBase) used as an outgroup. Numbers on the branches are the robustness (local bootstraps) as implemented in FastTree. One group (purple) may be at the origin of the 2 MITEs families (based on TIR similarities). The resulting tree suggests a monophyletic origin of the *C. congregata* Sola2 sequences. Our knowledge of Sola2 elements remains fragmentary, only few genomes having been screened. Closest sequences in Repbase are found in the related parasitoid *Nasonia vitripennis,* and two ant species. Potentially active sequences (long ORFs, no stop codons or frameshift, with one intron) could be identified in clusters 6 and 16.

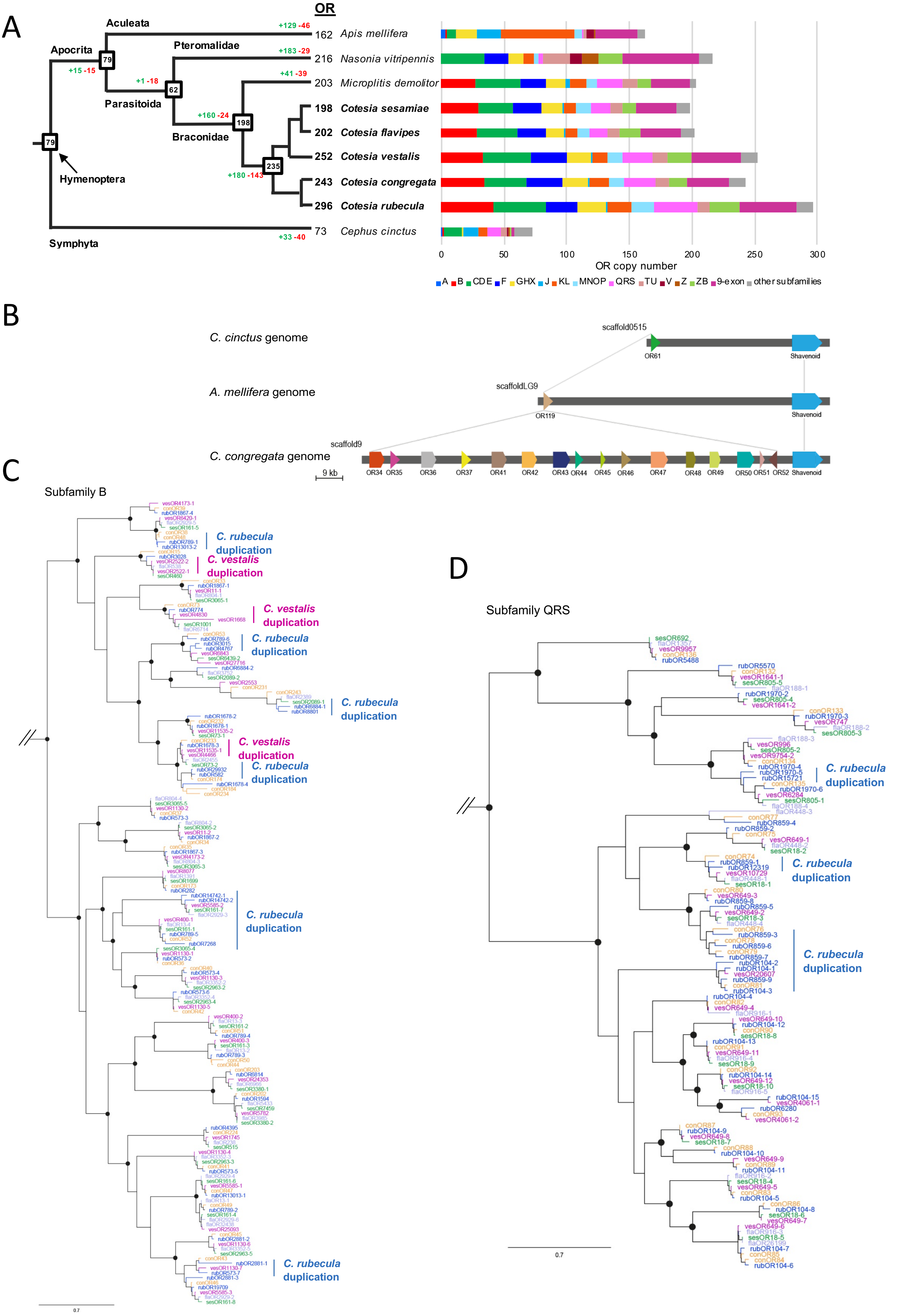
A
OR
Aculeata
Apocrita
Pteromalidae
Parasitoida
Braconidae
Hymenoptera
Symphyta
+129 -46
+183 -29
+41 -39
+15 -15
+1 -18
+160 -24
+180 -143
+33 -40
79
79
62
198
235
162 Apis mellifera
216 Nasonia vitripennis
203 Microplitis demolitor
198 Cotesia sesamiae
202 Cotesia flavipes
252 Cotesia vestalis
243 Cotesia congregata
296 Cotesia rubecula
73 Cephus cinctus
0 50 100 150 200 250 300
OR copy number
A B CDE F GHX J KL MNOP QRS TU V Z ZB 9-exon other subfamilies
B
C. cinctus genome
A. mellifera genome
C. congregata genome
scaffold0515
scaffoldLG9
scaffold9
OR61
Shavenoid
OR119
Shavenoid
OR34 OR35 OR36 OR37 OR41 OR42 OR43 OR44 OR45 OR46 OR47 OR48 OR49 OR50 OR51 OR52 Shavenoid
9 kb
C
Subfamily B
C. rubecula duplication
C. vestalis duplication
C. vestalis duplication
C. rubecula duplication
C. rubecula duplication
C. vestalis duplication
C. rubecula duplication
C. rubecula duplication
C. rubecula duplication
0.7
D
Subfamily QRS
C. rubecula duplication
C. rubecula duplication
C. rubecula duplication
0.7

Supplementary Figure 3. A Copy number dynamics of OR genes in five Cotesia species and four other Hymenoptera species. Estimated numbers of gene gain and loss events are shown on each branch of the species tree in green and red, respectively. The size of OR repertoires in common ancestors is indicated in boxes at the corresponding nodes of the species tree. The histogram represents the distribution of OR copy number per subfamily for each species. **B** Synteny among the OR subfamily B in *C. congregata*, *C. cinctus* and *A. mellifera*, based on inter-specific conservation of the shavenoid gene, showing expansion through tandem duplications for *C. congregate* OR genes. Orientation (arrows) of genes within scaffolds are indicated. **C** and **D** phylogenies OR subfamilies B and QRS respectively in five *Cotesia* species. These subtrees were extracted from maximum-likelihood phylogeny of *Cotesia* ORs including OR repertoires from *C. congregata* (orange), *C. flavipes* (purple), *C. rubecula* (blue), *C. sesamiae* (green) and *C. vestalis* (pink). The tree was rooted using the Orco clade as outgroup. Circles indicate nodes strongly supported by the approximate likelihood-ratio test (aLRT $\geq$ 0.95). The scale bar represents 0.7 expected amino acid substitutions per site. *C. rubecula* and *C. vestalis* duplicated gene copies are indicated on the right.

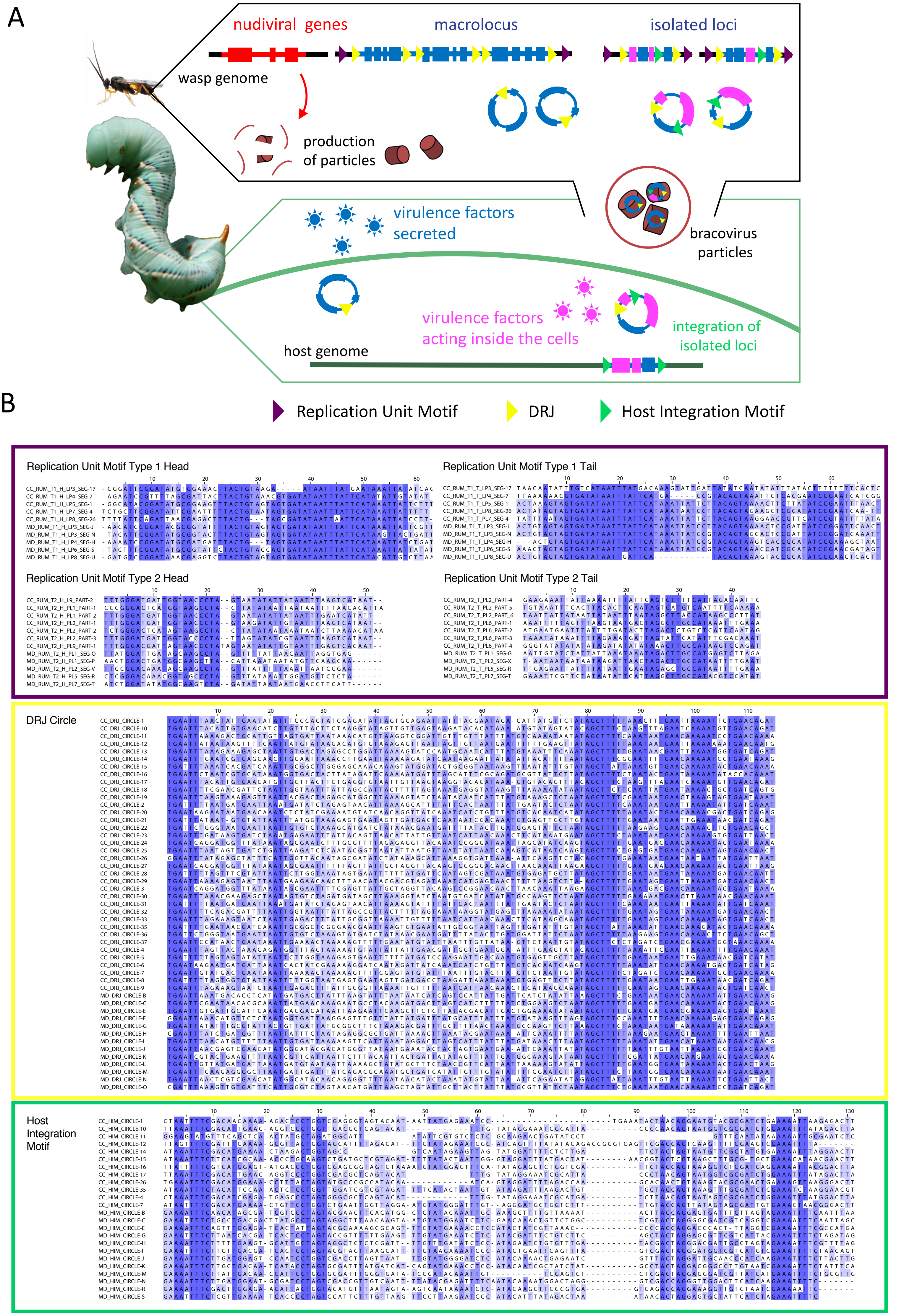
A
nudiviral genes
macrolocus
isolated loci
wasp genome
production of particles
bracovirus particles
virulence factors secreted
virulence factors acting inside the cells
integration of isolated loci
host genome
B
Replication Unit Motif
DRJ
Host Integration Motif
Replication Unit Motif Type 1 Head
Replication Unit Motif Type 1 Tail
Replication Unit Motif Type 2 Head
Replication Unit Motif Type 2 Tail
DRJ Circle
Host Integration Motif

Supplementary Figure 4. A Schematic representation of the bracovirus production in wasp ovaries and their function in host cells. Circles from isolated loci, encoding in particular *ptp* and *Vank* genes, integrate into parasitized host DNA using HIM site mediated mechanism. **B** In purple framework, the alignment of Replication Unit Motifs (RUM) including *C. congregata* and *M. demolitor* sequences. In yellow framework, the alignment of the circle Direct Repeat Junctions (DRJ). In green framework, the alignment of the Host Integration Motifs (HIM).

A

Protein fonction	Gene name	Gene content by species							dN/dS value	p-value
		C.c	*C.r*	*C.g*	*C.v*	*C.f*	*C.s*	*M.d*		
Replication, DNA processing	*helicase*	+	+	+	+	+	+	+	0.1343	< 0.001
	int_1	+	+	+	+	+	+	+	0.15141	< 0.001
	int_2	+	+	+	+	+	+	+	0.13921	< 0.001
	fen-1-1a	+	2	+	-	-	-	-	0.75876	0.284
	fen-1-1b	+	+	-	-	-	-	-	n.c.	n.c.
	fen-1-2	+	+	+	+	+	+	+	0.27467	< 0.001
	fen-1-3a	+	+	+	+	+	+	-	0.13039	< 0.001
	fen-1-3b	+	+	+	-	-	-	-	0.87961	0.555
	fen-1-3c	+	+	+	-	-	-	-	0.38875	< 0.001
Transcription	*p47*	+	+	+	+	+	+	+	0.05605	< 0.001
	lef-8	+	+	+	+	+	+	+	0.05973	< 0.001
	lef-9	+	+	+	+	+	+	+	0.05088	< 0.001
	lef-4	+	+	+	+	+	+	+	0.08525	< 0.001
	lef-5	+	+	+	+	+	+	+	0.36112	< 0.001
Packaging, assembly and release	*vlf-1*	+	+	+	+	+	+	+	0.17119	< 0.001
	vp91	+	+	+	+	+	+	+	0.3808	< 0.001
	vp39	+	+	+	+	+	+	+	0.72163	0.057
	p33_1	+	+	+	+	+	+	+	0.17862	< 0.001
	p33_2	2	1	1	1	1	1	1	0.15432*	< 0.001
	38K	+	+	+	+	+	+	+	0.19847	< 0.001
	p6.9_1	+	+	+	+	+	+	+	0.08337	< 0.001
	p6.9_2	+	+	+	+	+	+	+	0.70662	0.267
per os infectivity factors and ODV envelope particle components	*p74*	+	+	+	+	+	+	+	0.79274	0.058
	pif-1	+	+	+	+	+	+	+	0.45494	< 0.001
	pif-2	+	+	+	+	+	+	+	0.319	< 0.001
	pif-3	+	+	+	+	+	+	+	0.71855	0.098
	pif-4	+	+	+	+	+	+	+	0.45842	< 0.001
	pif-5_1	+	+	+	+	+	+	+	1.10226	0.38
	pif-5_2	2	1	1	1	1	1	3	0.3709*	< 0.001
	pif-6	+	+	+	+	+	+	+	0.41774	< 0.01
	HzNVorf9_1	+	+	+	+	+	+	+	0.08198	< 0.001
	HzNVorf9_2	+	+	+	+	+	+	+	0.08558	< 0.001
	GbNVorf19	+	+	+	+	+	+	+	0.30979	< 0.001
	HzNVorf64	4	1	1	1	1	1	1	0.09716*	< 0.001
	HzNVorf94	+	+	+	+	+	+	+	0.11672	< 0.001
	HzNVorf106	+	+	+	+	+	+	+	0.08689	< 0.001
	PmV	+	+	+	+	+	+	+	0.11206	< 0.001
	11K	+	+	+	+	+	+	+	0.29473	< 0.001
	HzNVorf128	+	+	+	+	+	+	+	0.11851	< 0.001
	HzNVorf140_1	3	1	1	1	1	1	1	0.19362*	< 0.001
	HzNVorf140_2	+	+	+	+	+	+	+	0.1557	< 0.001
	PmNVorf87	+	+	+	+	+	+	+	0.34099	< 0.001
	ToNVorf54_1	+	+	+	+	+	+	+	0.245	< 0.001
	ToNVorf54_2	+	+	+	+	+	+	+	0.23574	< 0.001
Other particle components	*17a_1*	+	+	+	+	+	+	+	0.96093	0.839
	17a_2	+	+	+	+	+	+	-	0.62831	< 0.05
	17b	+	+	+	+	+	+	+	0.11694	< 0.001
	27a	+	+	+	+	+	+	+	0.2348	< 0.001
	27b	+	+	+	+	+	+	+	0.24099	< 0.001
	30b	+	+	+	+	+	+	+	0.46248	< 0.001
	35a_1	+	+	+	+	+	+	+	0.74071	0.088
	35a_2	+	+	+	+	+	+	+	0.55692	< 0.001
	58b	+	+	+	+	+	+	+	0.58867	< 0.001
Unknown	*K425_438*	+	+	+	+	+	+	+	0.66340	0.076
	K425_445	+	+	+	+	+	+	+	0.83681	0.45
	K425_456	+	+	+	+	+	+	+	0.14541	< 0.001
	K425_459	+	+	+	+	+	+	+	0.20317	< 0.001
	K425_461	+	+	+	+	+	+	+	0.14772	< 0.001
	ToNVorf29	+	+	+	+	+	+	+	0.15643	< 0.001
	HzNVorf93	+	+	+	+	+	+	+	0.18237	< 0.001
	OrNVorf18	+	+	+	+	+	+	+	0.36808	< 0.001
odv-e66	*odv-e66-1*	+	+	+	+	+	+	-	0.57915	< 0.001
	odv-e66-2	+	+	+	+	+	+	+	0.84754	< 0.001
	odv-e66-3	+	+	+	+	+	+	+	0.51583	< 0.01
	odv-e66-4	+	+	+	+	+	+	-	0.53930	< 0.001
	odv-e66-5	+	+	+	+	+	+	-	0.71140	< 0.05
	odv-e66-6/7/8/9	4	1	1	3	1	1	2	n.c.	n.c.
	odv-e66-10	+	+	-	+	+	+	-	1.10867	0.589
	odv-e66-11	+	+	+	+	+	+	-	0.25798	< 0.001
	odv-e66-12	+	+	+	+	+	+	-	0.23769	< 0.001
	odv-e66-13	+	+	+	+	+	+	+	0.87336	0.439
	odv-e66-14	+	+	+	+	+	+	+	0.61298	< 0.01
	odv-e66-15	+	+	+	+	+	+	+	0.25491	< 0.001
	odv-e66-16	+	+	+	+	+	+	+	0.51120	< 0.001
	odv-e66-17	+	+	+	+	+	+	+	0.58245	< 0.001
	odv-e66-18	+	+	+	+	+	+	-	0.41734	< 0.001
	odv-e66-19	+	+	+	+	+	+	+	0.49926	< 0.001
	odv-e66-20	+	+	+	+	+	+	+	0.17390	< 0.001
	odv-e66-21/22/23/ 24/25/26/27/28	8	1	-	-	-	-	-	n.c.	n.c.
	odv-e66-29	+	+	+	+	+	+	2	0.36489	< 0.001
	odv-e66-30	+	+	+	+	+	+	-	0.85965	0.211
	odv-e66-31	+	+	-	-	-	-	-	n.c.	n.c.
	odv-e66-32	+	+	-	+	+	+	-	0.52025	< 0.001
	odv-e66-33	+	+	-	-	-	-	-	n.c.	n.c.
	odv-e66-34	+	+	-	-	-	-	-	n.c.	n.c.
	odv-e66-35	+	-	-	-	-	-	-	n.c.	n.c.
	odv-e66-36	+	-	-	-	-	-	-	n.c.	n.c.

* the closest orthologue among *C.congregata* duplications was used to estimate dN/dS
n.c. - dN/dS was not etimated when orthologues and paralogues were not distinguishable

B

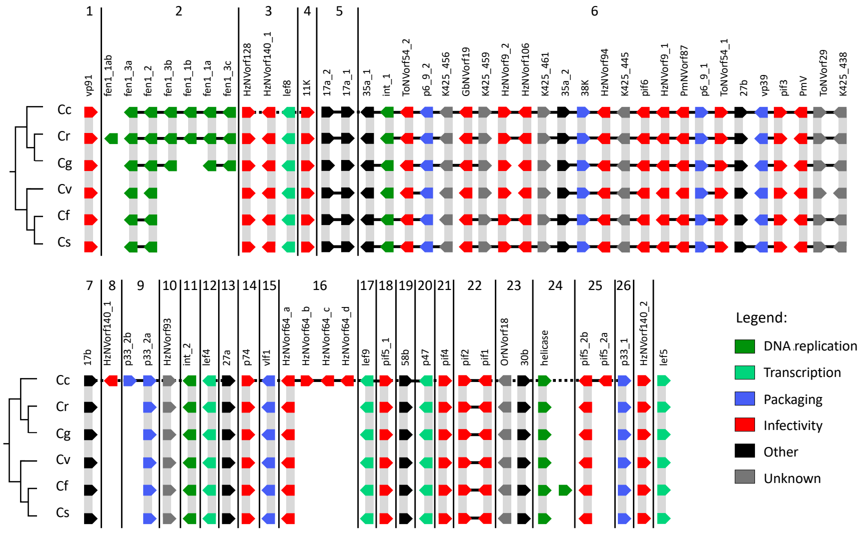

Supplementary Figure 5. A Evolutionary rates of nudiviral genes in *Cotesia* **B** Synteny of nudiviral genes across *Cotesia* species. Continuous black lines represent scaffolds and arrows indicate the orientation of the genes in each species. Number 1 to 26 correspond to the 26 nudiviral loci identified in *C. congregata* chromosomes. species (*C.c* = *C. congregata*; *C.r* = *C. rubecula*; *C.g* = *C. glomerata*; *C.v* = *C. vestalis*; *C.f* = *C. flavipes*; *C.s* = *C. sesamiae*; *M.d* = *M. demolitor.*

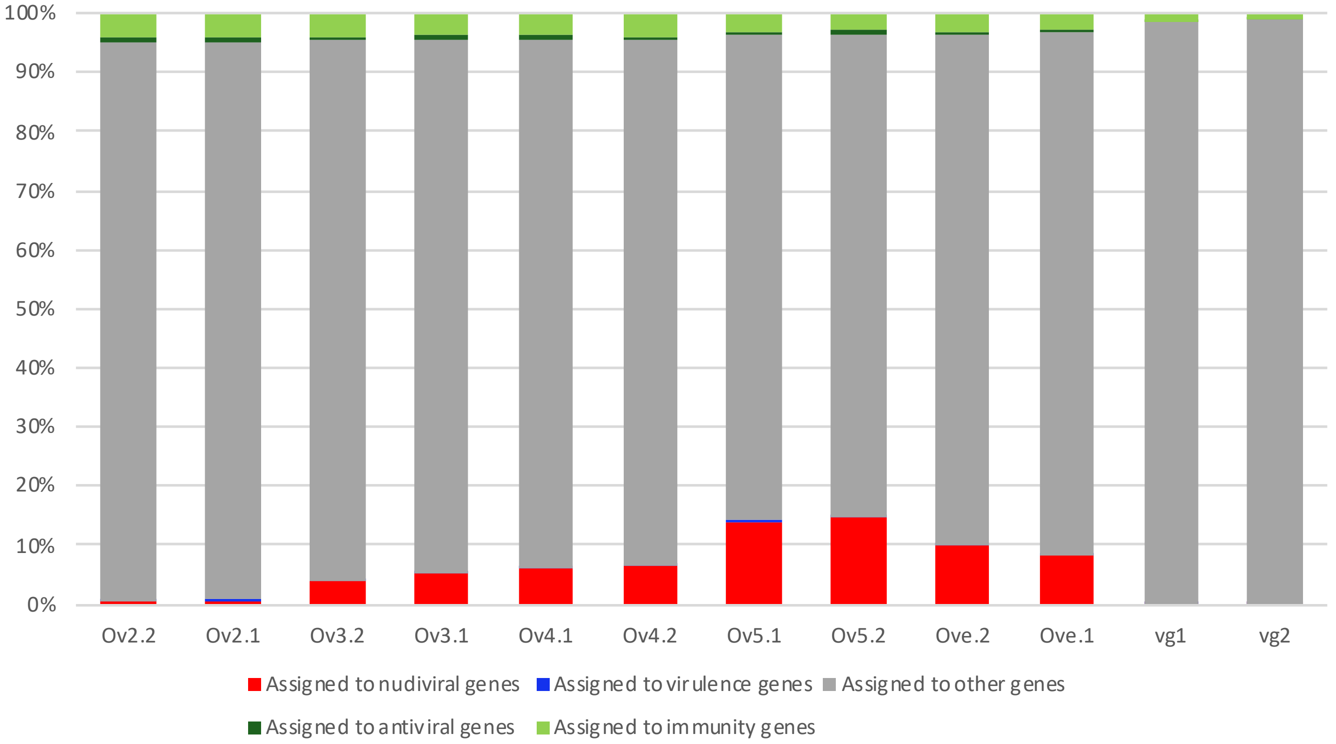
100%
90%
80%
70%
60%
50%
40%
30%
20%
10%
0%
Ov2.2
Ov2.1
Ov3.2
Ov3.1
Ov4.1
Ov4.2
Ov5.1
Ov5.2
Ove.2
Ove.1
vg1
vg2
Assigned to nudiviral genes
Assigned to virulence genes
Assigned to other genes
Assigned to antiviral genes
Assigned to immunity genes

Supplementary Figure 6. Percentage of reads assigned to nudiviral, virulence or other genes out of the total number of reads assigned to *C. congregata* gene features.

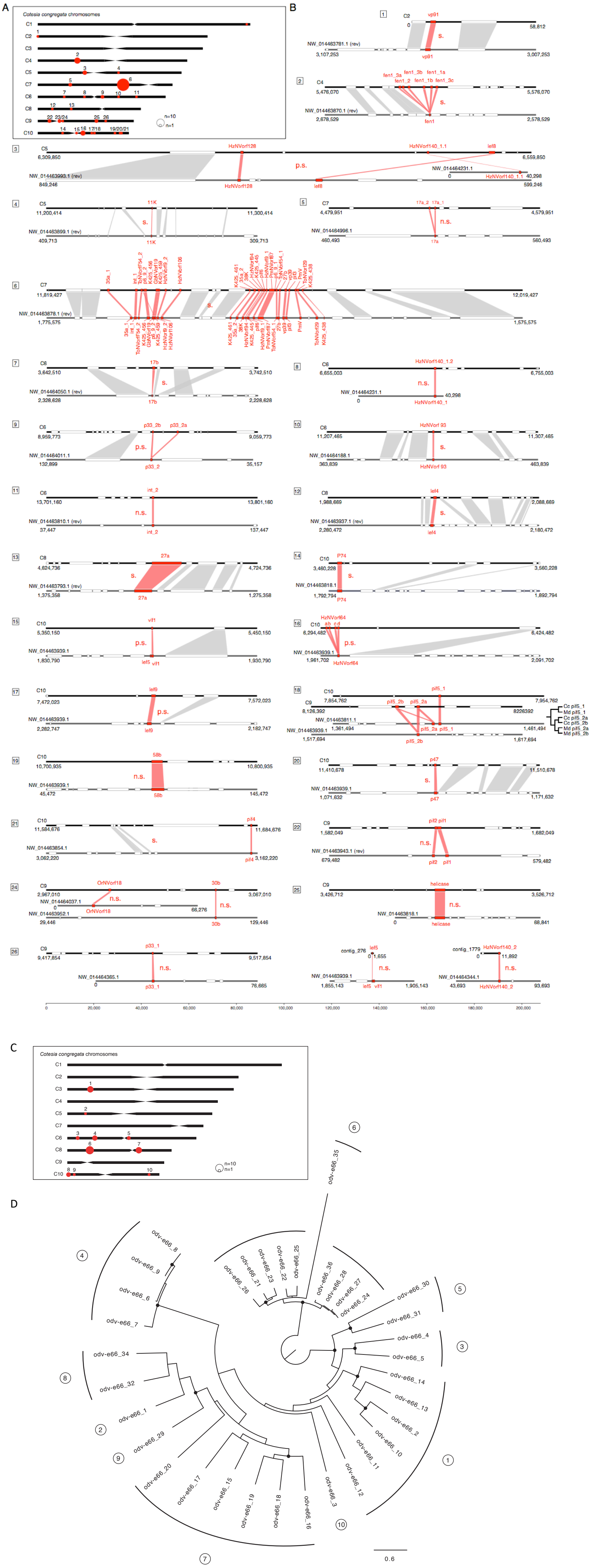

A
Cotesia congregata chromosomes
C1
C2
C3
C4
C5
C7
C6
C8
C9
C10
n=10
n=1
B
C
Cotesia congregata chromosomes
n=10
n=1
D
0.6

Supplementary Figure 7. Synteny between nudiviral genes containing regions of *C. congregata* and *M. demolitor*. **A** *C. congregata* chromosomes map. **B** Comparison of nudiviral genes regions of *C. congregata* and *M. demolitor*. To validate a synteny between the two specie (indicated by "s." for synteny), we searched for at least two hymenopteran (non-viral) orthologous gene in the vicinity of homologous nudiviral gene(s) of the two species. If only one non-nudiviral orthologous gene was present we considered synteny as probable ("p.s.": probable synteny). Finally, when no orthologous gene was present in the vicinity of the nudiviral gene(s) in the two species, we considered the regions containing nudiviral genes were not homologous ("n.s": no synteny). *C. congregata* chromosomes and *M. demolitor* genome scaffolds are represented in black and dark grey respectively. Numbers 1 to 26 correspond to the 26 loci containing nudiviral genes identified in *C. congregata* chromosomes map. Red boxes indicate nudiviral genes and white boxes refer to hymenopteran genes. *M. demolitor* scaffolds for which the orientation is reversed compared to *C. congregata* chromosomes are indicated by "rev". The scale shows length in bp. **C** Localization of nudiviral odv-e66 genes on *C. congregata* chromosomes. The 36 odv-e66 genes are clustered in 10 groups distributed in five different chromosomes of *C. congregata*. **D** Maximum-likelihood phylogeny of *C. congregata* odv-e66 family (1,000 bootstraps). Prior tree construction, the regions that were present in less than 50% of the aligned sequences were manually curated from the codon-based alignment and the odv-e66_33 gene was excluded due to its short sequence. The tree was rooted to the midpoint and the black dots indicate nodes with at least 80% of support.

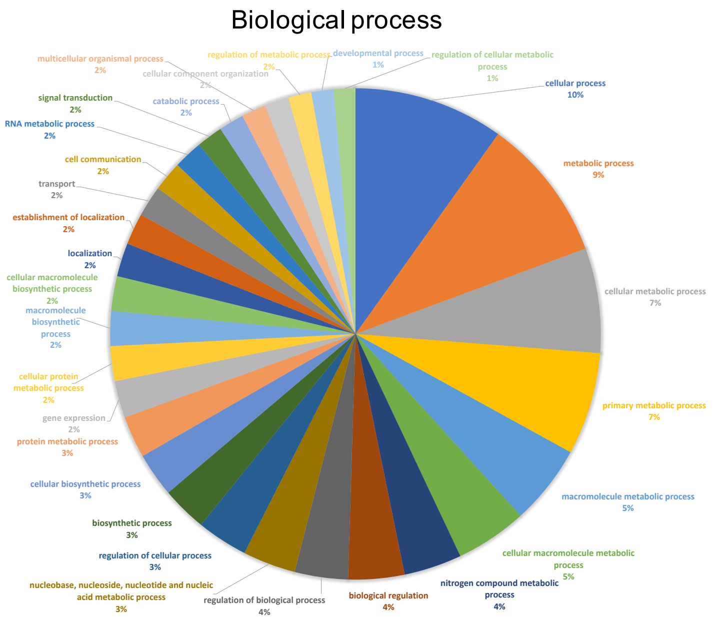
Biological process
multicellular organismal process 2%
regulation of metabolic process 2%
developmental process 1%
regulation of cellular metabolic process 1%
cellular component organization 2%
catabolic process 2%
signal transduction 2%
RNA metabolic process 2%
cell communication 2%
transport 2%
establishment of localization 2%
localization 2%
cellular macromolecule biosynthetic process 2%
macromolecule biosynthetic process 2%
cellular protein metabolic process 2%
gene expression 2%
protein metabolic process 3%
cellular biosynthetic process 3%
biosynthetic process 3%
regulation of cellular process 3%
nucleobase, nucleoside, nucleotide and nucleic acid metabolic process 3%
regulation of biological process 4%
biological regulation 4%
nitrogen compound metabolic process 4%
cellular macromolecule metabolic process 5%
macromolecule metabolic process 5%
primary metabolic process 7%
cellular metabolic process 7%
metabolic process 9%
cellular process 10%

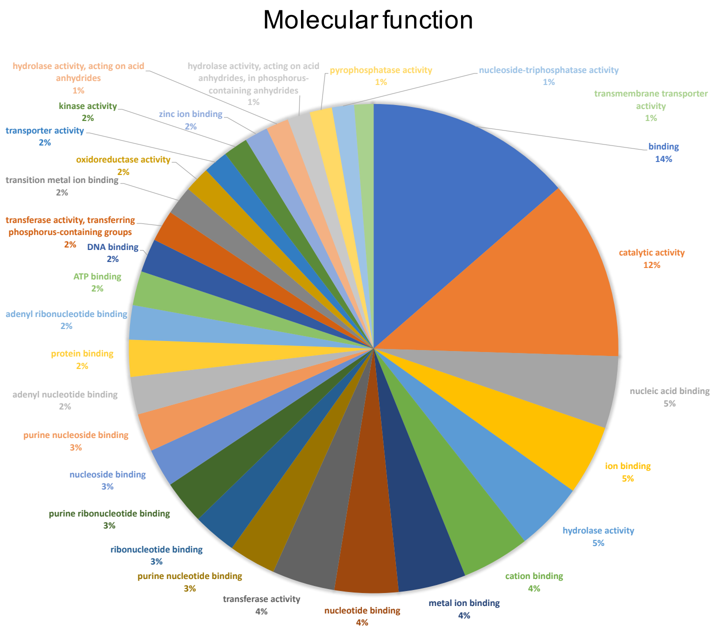
Molecular function
hydrolase activity, acting on acid anhydrides 1%
hydrolase activity, acting on acid anhydrides, in phosphorus-containing anhydrides 1%
pyrophosphatase activity 1%
nucleoside-triphosphatase activity 1%
transmembrane transporter activity 1%
kinase activity 2%
zinc ion binding 2%
transporter activity 2%
oxidoreductase activity 2%
transition metal ion binding 2%
transferase activity, transferring phosphorus-containing groups 2%
DNA binding 2%
ATP binding 2%
adenyl ribonucleotide binding 2%
protein binding 2%
adenyl nucleotide binding 2%
purine nucleoside binding 3%
nucleoside binding 3%
purine ribonucleotide binding 3%
ribonucleotide binding 3%
purine nucleotide binding 3%
transferase activity 4%
nucleotide binding 4%
metal ion binding 4%
cation binding 4%
hydrolase activity 5%
ion binding 5%
nucleic acid binding 5%
catalytic activity 12%
binding 14%

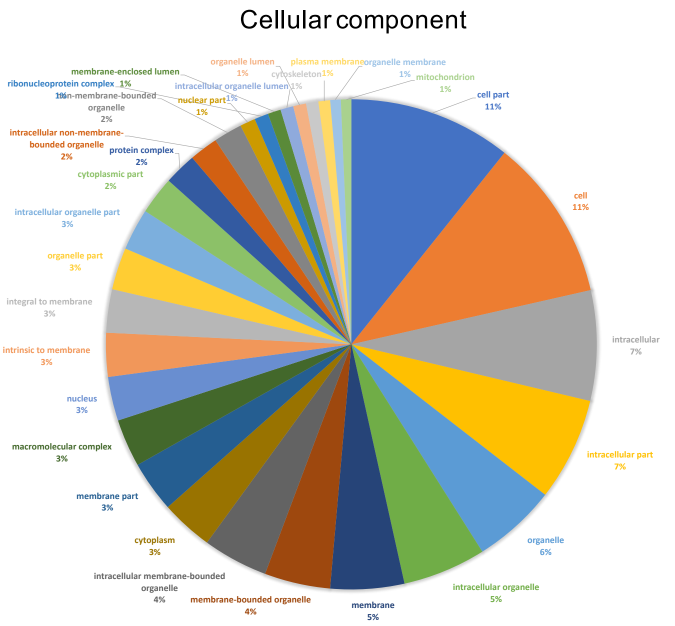
Cellular component
organelle lumen 1%
plasma membrane 1%
organelle membrane 1%
membrane-enclosed lumen 1%
cytoskeleton 1%
mitochondrion 1%
ribonucleoprotein complex 1%
intracellular organelle lumen 1%
non-membrane-bounded organelle 2%
nuclear part 1%
cell part 11%
intracellular non-membrane-bounded organelle 2%
protein complex 2%
cytoplasmic part 2%
intracellular organelle part 3%
organelle part 3%
integral to membrane 3%
intrinsic to membrane 3%
nucleus 3%
macromolecular complex 3%
membrane part 3%
cytoplasm 3%
intracellular membrane-bounded organelle 4%
membrane-bounded organelle 4%
membrane 5%
intracellular organelle 5%
organelle 6%
intracellular part 7%
intracellular 7%
cell 11%

Supplementary Figure 8. Pie chart representations of gene ontology (GO) annotation results of *C. congregata* genome. GO hits were assigned to biological process, molecular function and cellular component. To facilitate visualization, only the 30 most abundant GO terms are shown for each category.

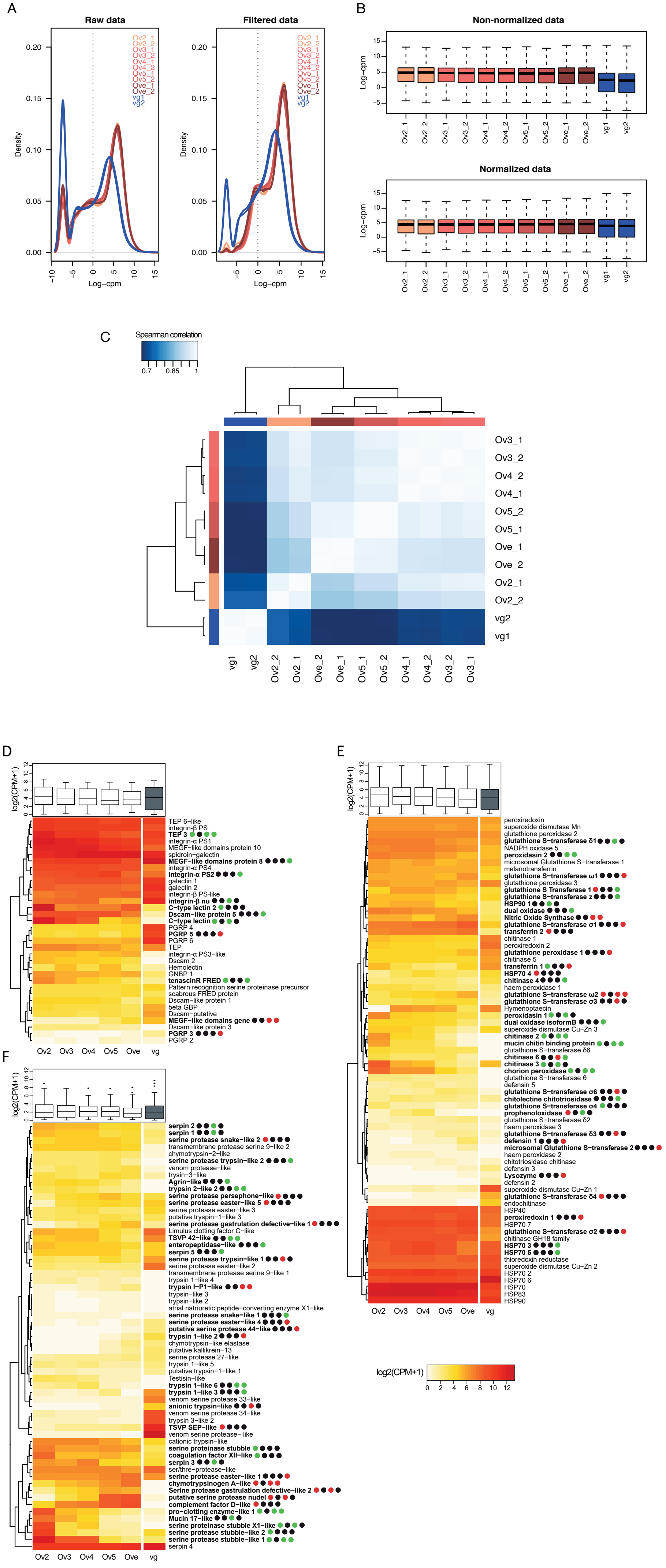
A
Raw data
Filtered data
Density
Log−cpm
Ov2_1
Ov2_2
Ov3_1
Ov3_2
Ov4_1
Ov4_2
Ov5_1
Ov5_2
Ove_1
Ove_2
vg1
vg2
B
Non-normalized data
Normalized data
Log−cpm
C
Spearman correlation
0.7
0.85
1
Ov3_1
Ov3_2
Ov4_2
Ov4_1
Ov5_2
Ov5_1
Ove_1
Ove_2
Ov2_1
Ov2_2
vg2
vg1
D
log2(CPM+1)
TEP 6−like
integrin-β PS
TEP 3
integrin-α PS1
MEGF−like domains protein 10
spidroin−galectin
MEGF−like domains protein 8
integrin-α PS4
integrin-α PS2
galectin 1
galectin 2
integrin-β PS-like
integrin-β nu
C−type lectin 2
Dscam−like protein 5
C−type lectin
PGRP 4
PGRP 5
PGRP 6
TEP
integrin-α PS3−like
Dscam 2
Hemolectin
GNBP 1
tenascinR FRED
Pattern recognition serine proteinase precursor
scabrous FRED protein
Dscam−like protein 1
beta GBP
Dscam−putative
MEGF−like domains gene
Dscam−like protein 3
PGRP 3
PGRP 2
Ov2
Ov3
Ov4
Ov5
Ove
vg
E
log2(CPM+1)
peroxiredoxin
superoxide dismutase Mn
glutathione peroxidase 2
glutathione S−transferase δ1
NADPH oxidase 5
peroxidasin 2
microsomal Glutathione S−transferase 1
melanotransferrin
glutathione S−transferase ω1
glutathione peroxidase 3
glutathione S Transferase 1
glutathione S−transferase z
HSP90 1
dual oxidase
Nitric Oxide Synthase
glutathione S−transferase σ1
transferrin 2
chitinase 1
peroxiredoxin 2
glutathione peroxidase 1
chitinase 5
transferrin 1
HSP70 4
chitinase 4
haem peroxidase 1
glutathione S−transferase ω2
glutathione S−transferase σ3
Hymenoptaecin
peroxidasin 1
dual oxidase isoformB
superoxide dismutase Cu−Zn 3
chitinase 2
mucin chitin binding protein
glutathione S−transferase δ6
chitinase 6
chitinase 3
chorion peroxidase
glutathione S−transferase θ
defensin 5
glutathione S−transferase σ6
chitolectine chitotriosidase
glutathione S−transferase σ4
prophenoloxidase
glutathione S−transferase δ2
haem peroxidase 3
glutathione S−transferase δ3
defensin 1
microsomal Glutathione S−transferase 2
haem peroxidase 2
chitotriosidase chitinase
defensin 3
Lysozyme
defensin 2
superoxide dismutase Cu−Zn 1
glutathione S−transferase δ4
endochitinase
HSP40
peroxiredoxin 1
HSP70 7
glutathione S−transferase σ2
chitinase GH18 family
HSP70 3
HSP70 5
thioredoxin reductase
superoxide dismutase Cu−Zn 2
HSP70 2
HSP70 6
HSP70
HSP83
HSP90
Ov2
Ov3
Ov4
Ov5
Ove
vg
log2(CPM+1)
0
2
4
6
8
10
12
F
log2(CPM+1)
serpin 2
serpin 1
serine protease snake−like 2
transmembrane protease serine 9−like 2
chymotrypsin−2−like
serine protease trypsin−like 2
venom protease−like
trysin−3−like
Agrin−like
trypsin 2−like 2
serine protease persephone−like
serine protease easter−like 5
serine protease easter−like 3
putative tryspin−1−like 3
serine protease gastrulation defective−like 1
Limulus clotting factor C−like
TSVP 42−like
enteropeptidase−like
serpin 5
serine protease trypsin−like 1
serine protease easter−like 2
transmembrane protease serine 9−like 1
trypsin 1−like 4
trypsin I−P1−like
trypsin−like 3
trypsin−like 2
atrial natriuretic peptide−converting enzyme X1−like
serine protease snake−like 1
serine protease easter−like 4
putative serine protease 44−like
trypsin 1−like 2
chymotrypsin−like elastase
putative kallikrein−13
serine protease 27−like
trypsin 1−like 5
putative trypsin−1−like 1
Testisin−like
trypsin 1−like 6
trypsin 1−like 3
venom serine protease 33−like
anionic trypsin−like
venom serine protease 34−like
trypsin 3−like 2
TSVP SEP−like
venom serine protease− like
cationic trypsin−like
serine proteinase stubble
coagulation factor XII−like
serpin 3
ser/thre−protease−like
serine protease easter−like 1
chymotrypsinogen A−like
Serine protease gastrulation defective−like 2
putative serine protease nudel
complement factor D−like
pro−clotting enzyme−like 1
Mucin 17−like
serine proteinase stubble X1−like
serine protease stubble−like 2
serine protease stubble−like 1
serpin 4
Ov2
Ov3
Ov4
Ov5
Ove
vg

Supplementary Figure 9. Gene expression analysis of ovaries and venom glands of *Cotesia congregata.* **A** Density plots representing expression levels of all expressed genes (Raw data: 13,607 genes) and after filtering the genes that do not show CPM > 0.4 in at least 2 libraries on the 12 analyzed libraries (Filtered data: 11,216 genes). **B** Expression level distribution of the genes (filtered data) before and after CPM normalization using TMM method in edgeR. **C** Heatmap of Spearman correlation between the 12 analyzed libraries. The unsupervised clustering did not reveal discrepancies between biological replicates, then all libraries were retained for further analyses. Ov2, Ov3, Ov4 and Ov5 represent ovary samples collected at different larval stages. Ove and vg respectively refer to ovaries and venom glands from adult wasp. Heatmaps show the expression of the genes involved in **D** signal recognition, **E** signal transduction and **F** effector functions across the developmental stages of ovaries (Ov2, Ov3, Ov4, Ov5, Ove) and in venom glands (vg). The trees on the left are unsupervised hierarchical clustering of expression values. Boxplots represent overall expression of each immunity gene group in ovaries and venom glands. Bold names highlight the genes that are differentially expressed and dots represent the four different comparisons studied between consecutive ovary stages (Ov2 *vs.* Ov3, Ov3 *vs*. Ov4, Ov4 *vs*. Ov5 and Ov5 *vs.* Ove). Black, red and green dots indicate similar, increased and reduced expressions between consecutive developmental stages respectively.
