# Supplementary material for: Chromosomal scale assembly of parasitic wasp genome reveals symbiotic virus colonization
Source: Commun Biol. 2021 Jan 22;4:104. doi: 10.1038/s42003-020-01623-8 (PMC7822920; doi:10.1038/s42003-020-01623-8)
Supplement: Supplementary file 3 — Description of Additional Supplementary Files [file 42003_2020_1623_MOESM3_ESM.pdf]

Description of Additional Supplementary Files

File name: Supplementary Data 1.

Description: Sequencing, assembly and annotation statistics of the six Cotesia genomes. These statstics include results of BUSCO analyses on genomes and predicted proteins performed to assess genomes and annotations completeness.

File name: Supplementary Data 2.

Description: Gene repertories of the Cotesia physiological functions studied: olfaction, detoxification and immunity. Gene numbers of insect species at different phylogenetic levels are indicated for comparisons according to available data in the literature. Values in bold correspond to genes newly annotated in this study by experts of genes involved in insect olfaction detoxification or immunity (in C. congregata only or in all species depending on the function). n.d. : not determined; - : data not available or not relevant (for example OR subfamilies are not conserved outside Hymenoptera). Cc = C. congregata; Cr = C. rubecula; Cg = C. glomerata; Cv = C. vestalis; Cf = C. flavipes; Cs = C. sesamiae; Md = M. demolitor.

File name: Supplementary Data 3.

Description: Normalized expression levels of C. congregata annotated genes during ovary development. The columns correspond to automated gene name, gene category, gene family, annotation (automated or manual) followed by gene expression levels indicated in log2[Counts Per Million (CPM)+1] for the six studied stages. Ov2, Ov3, Ov4, Ov5 and Ove represent ovary samples collected at different pupal stages from day 2 to day 5 after cocoon spinning and Ove and vg respectively refer to ovaries and venom glands from adult wasp just emerged. The nudiviral genes are highlighted in red and “nc” in the "Gene category" indicates that the gene belongs to the Nudiviral Cluster. NE indicates genes that were not expressed in any tissue.

File name: Supplementary Data 4.

Description: Mapping statistics from Cotesia congregata ovary (Ov) and venom gland (vg) RNAseq libraries.
