# Supplementary material for: Chromosomal scale assembly of parasitic wasp genome reveals symbiotic virus colonization
Source: Commun Biol. 2021 Jan 22;4:104. doi: 10.1038/s42003-020-01623-8 (PMC7822920; doi:10.1038/s42003-020-01623-8)
Supplement: Supplementary file 5 — Reporting Summary [file 42003_2020_1623_MOESM5_ESM.pdf]

nature research

Corresponding author(s): NCOMMS-20-11743-T
drezen@univ-tours.fr

Last updated by author(s): Dec 3, 2020

Reporting Summary

Nature Research wishes to improve the reproducibility of the work that we publish. This form provides structure for consistency and transparency in reporting. For further information on Nature Research policies, see Authors & Referees and the Editorial Policy Checklist .

Statistics

For all statistical analyses, confirm that the following items are present in the figure legend, table legend, main text, or Methods section.

n/a	Confirmed	
☐	☒	The exact sample size (*n*) for each experimental group/condition, given as a discrete number and unit of measurement
☐	☒	A statement on whether measurements were taken from distinct samples or whether the same sample was measured repeatedly
☐	☒	The statistical test(s) used AND whether they are one- or two-sided<br>*Only common tests should be described solely by name; describe more complex techniques in the Methods section.*
☒	☐	A description of all covariates tested
☐	☒	A description of any assumptions or corrections, such as tests of normality and adjustment for multiple comparisons
☐	☒	A full description of the statistical parameters including central tendency (e.g. means) or other basic estimates (e.g. regression coefficient) AND variation (e.g. standard deviation) or associated estimates of uncertainty (e.g. confidence intervals)
☐	☒	For null hypothesis testing, the test statistic (e.g. *F*, *t*, *r*) with confidence intervals, effect sizes, degrees of freedom and *P* value noted<br>*Give P values as exact values whenever suitable.*
☒	☐	For Bayesian analysis, information on the choice of priors and Markov chain Monte Carlo settings
☒	☐	For hierarchical and complex designs, identification of the appropriate level for tests and full reporting of outcomes
☒	☐	Estimates of effect sizes (e.g. Cohen's *d*, Pearson's *r*), indicating how they were calculated

Our web collection on statistics for biologists contains articles on many of the points above.

Software and code

Policy information about availability of computer code

Data collection	No software was used for data collections, except those built in the 454, illumina and Pacbio next generation sequencers.
Data analysis	Publicly-available software used in this study includes: Newbler, fastxtend, FASTX-Toolkit, SOAPdenovo, Velvet, Jellyfish, HiCbox, instaGRAAL, QUAST-LG, BUSCO, REPET, Gmove, SNAP, MAKER, BLAST+, Diamond, Blast2GO, bowtie2, Blobtools, MAFFT, PhyML, jModelTest, NOTUNG, MACSE, PAML, TopHat and edgeR. The tools, their version as well as the parameters used to analyze the data are reported in details the material and methods section. Custom scripts are available on this github : https://github.com/JeremyLGauthier/Scripts_Cotesia_Genomes

For manuscripts utilizing custom algorithms or software that are central to the research but not yet described in published literature, software must be made available to editors/reviewers. We strongly encourage code deposition in a community repository (e.g. GitHub). See the Nature Research guidelines for submitting code & software for further information.

Data

Policy information about availability of data

All manuscripts must include a data availability statement. This statement should provide the following information, where applicable:

- Accession codes, unique identifiers, or web links for publicly available datasets
- A list of figures that have associated raw data
- A description of any restrictions on data availability

The datasets generated during the current study underlying genome sequencing and transcriptome analysis are available at the National Center for Biotechnology Information (NCBI): under the umbrella project PRJEB40240, comprising BioProject PRJEB36310 for genome raw reads, BioProject PRJNA594477 for transcriptome raw reads. A genome browser is available for each species on the web site BIPAA (Bioinformatic Platform for Agrosystem Arthropods, https://bipaa.genouest.org/is/parwaspdb). Chromosomal scale assembly of C. congregata genome is also available at BIPAA. All data have now been relesased publicly

Field-specific reporting

Please select the one below that is the best fit for your research. If you are not sure, read the appropriate sections before making your selection.

☐ Life sciences ☐ Behavioural & social sciences ☒ Ecological, evolutionary & environmental sciences

For a reference copy of the document with all sections, see nature.com/documents/nr-reporting-summary-flat.pdf

Ecological, evolutionary & environmental sciences study design

All studies must disclose on these points even when the disclosure is negative.

Study description	This paper describes the sequencing of the parasitoid wasp Cotesia congregata (assembly obtained at chromosomal scale) and five other Cotesia species. We studied the organisation and evolution of viral sequences since their integration in the wasp genome 100 million years ago by comparative genomics. We measured selection pressures operating on viral genes and used transcriptome data to study viral genes expression and antiviral immunity during wasp pupal development.
Research sample	Laboratory strains of Cotesia congregata, C. rubecula, C. glomerata, C. vestalis, C.flavipes, C.sesamiae reared in the different laboratories of the Cotesia consortium (see more details in the materials & methods section)
Sampling strategy	For genomic DNA sequencing, all DNA was extracted from strains reared in laboratories for multiple generations having low polymorphism. The process is described in greater detail in the supplemental Materials and Methods.
Data collection	Data was collected at the Genoscope (Evry, France) and University Medical Center of Groningen (Netherlands) using standard next-generation sequencers.The process is described in greater detail in the supplemental Materials and Methods.
Timing and spatial scale	DNA samples were collected independently by the different laboratories over several years and sent to sequencing centers when available. Cotesia congregata ovaries RNA samples were collected during a six month period.
Data exclusions	Fragmented genes from genome sobtained using Illumina only were not included in selection pressure measurments and phylogenetic analyses.
Reproducibility	Two independent iterations performed for chromosomal scale assembly gave only minor differences. Reproducibility between biological replicates in C. congregata RNA seq analysis was analysed and samples were not found statistically different.
Randomization	Randomization was not relevant to this study, as we were not testing for differences between populations.
Blinding	Blinding was not relevant to this study, as we were not testing for differences between populations.

Did the study involve field work? ☐ Yes ☒ No

Reporting for specific materials, systems and methods

We require information from authors about some types of materials, experimental systems and methods used in many studies. Here, indicate whether each material, system or method listed is relevant to your study. If you are not sure if a list item applies to your research, read the appropriate section before selecting a response.

Materials & experimental systems

n/a	Involved in the study
☒	☐ Antibodies
☒	☐ Eukaryotic cell lines
☒	☐ Palaeontology
☐	☒ Animals and other organisms
☒	☐ Human research participants
☒	☐ Clinical data

Methods

n/a	Involved in the study
☒	☐ ChIP-seq
☒	☐ Flow cytometry
☒	☐ MRI-based neuroimaging

Animals and other organisms

Policy information about studies involving animals; ARRIVE guidelines recommended for reporting animal research

Laboratory animals	Our animals are Cotesia congregata, C. rubecula, C. glomerata, C. vestalis, C.flavipes, C.sesamiae. They were originally caught in the wild and raised in laboratories for multiple years. C. congregata are raised in IRBI (Tours, France), C.glomerata C. rubecula and C. vestalis are raised at the University of Wageningen (Netherland) C. flavipes is raised at University of Sao Paulo (Brazil), C. sesamiae is raised at EGCE CNRS/IRD laboratory in Gif-sur-Yvette (France).

Wild animals	No wild animal was used
Field-collected samples	The study did not involve field collected animals.
Ethics oversight	To our knowledge no official rules apply regarding insects but all animals were anesthezised on ice before dissections.

Note that full information on the approval of the study protocol must also be provided in the manuscript.
